# Virtual reality in concussion management: from lab to clinic

**Published:** 2020-04-28

**Authors:** Fernando V. Santos, Felipe Yamaguchi, Thomas A. Buckley, Jaclyn B. Caccese

**Affiliations:** ^1^Bertec Corporation, Columbus, OH, United States; ^2^Department of Kinesiology and Applied Physiology, University of Delaware, Delaware, United States; ^3^School of Health and Rehabilitation Sciences, College of Medicine, The Ohio State University, Columbus, OH, United States

**Keywords:** Assessment, extended reality, neuropsychological testing, postural control, traumatic brain injury

## Abstract

The use of virtual reality (VR) technology continues to grow in the areas of clinical assessment and rehabilitation. Both researchers and health-care providers are exploring ways to incorporate VR in clinical practice as an emerging technology. VR postural control and neuropsychological testing represent a promising next step in sport-related concussion (SRC) management. This article reviews the current literature on VR applications for SRC assessment.

**Relevance for Patients:**

VR-based postural control assessments suggest that visual motion is destabilizing following SRC, perhaps indicating persistent perceptual-motion disintegration when clinical postural control tests suggest complete recovery. VR can also provide functional neuropsychological assessments using real-life scenarios or virtual environments, which may be more sensitive than traditional pencil-and-paper or computerized neuropsychological assessments.

## 1. Introduction

Extended reality (XR) is the use of all virtual and human-machine interactions. XR is the combination of virtual reality (VR), augmented reality (AR), and mixed reality (MR). VR can be defined as computer-generated simulated environments in real or imagined worlds [1-3]. In VR, the user is able to interact with the virtual environment without using visual cues from the real world, sometimes using digital recreations of his/her body (i.e., avatars) [1-3]. VR technologies include standard computer or television monitors, surround-screen displays, head-mounted displays, and dome-type projections [1,2]. VR technologies can be immersive or non-immersive, according to the sense of existence from the user [4,5]. Immersive VR technologies (e.g., head-mounted displays) project the user into a three-dimensional (3D) environment [4,5]. In non-immersive VR technologies (e.g., standard computer or television monitors), the user interacts with the simulated world using a device such as a controller or a computer mouse [4,5]. AR can be defined as the projection of virtual objects into the real world [1,3,6]. In AR, the user is not able to interact with the virtual overlay of digital information in the real world [1,3,6]. AR technologies most commonly include smartphones, although some head-mounted displays and/or smart glasses are available [1,3,6]. MR is a combination of VR and AR. In MR, the user can see the virtual object being projected into the real world and can interact with it [1,2]. For example, a user can touch or grab a virtual object using his/her own hand. Considering the rapidly evolving landscape of XR, VR, AR, and MR, we provide our working definitions of these technologies but acknowledge that there may be differing opinions.

There is growing interest in VR for traumatic brain injury (TBI) assessment and rehabilitation [7]. TBI can affect a number of different physical, cognitive, and behavioral functions and VR assessments and rehabilitation interventions can target these various domains. For example, one study assessed memory in TBI survivors and healthy age- and gender-matched controls with educational and occupational backgrounds similar to the TBI group [8]. Researchers created a virtual street, which included a low distraction zone and a high distraction zone. Distractions included both visual and auditory stimuli. Participants were asked to complete memory tasks while moving along the street (e.g., complete ten errands on a checklist). As expected, the TBI group performed worse on the memory tasks and were more affected by distractions [8]. In addition to studies examining cognitive performance in TBI survivors [8,9], other studies have examined target acquisition during reaching [10,11], postural control during standing and walking [12,13], and driving performance in individuals with mild to severe TBI [14]. Together, these studies suggest that VR has the advantage of assessing of complex sets of physical, cognitive, and behavioral functions rather than the isolated components assessed by traditional measures. This is particularly important in the understanding recovery from sport-related concussion (SRC). To date, clinical symptoms are still the best indicator of SRC diagnosis and recovery [15]. Furthermore, competitive athletes often have cognitive and motor reserve and may not be sufficiently challenged by current assessment protocols [16]. VR may allow for more challenging assessment protocols while still providing precise control and standardized presentation of task stimuli and outcome measures. This review will focus on the use of VR as an SRC assessment tool.

## 2. Enhancing SRC Management through VR

VR postural control and neuropsychological testing represent a potentially promising next step in SRC management. To be useful in a clinical setting, VR-based assessments must be ecologically valid and psychometrically equivalent to or better than traditional assessment tools. Several studies have examined the use of VR-based assessments in sub-acute SRC management (Table 1). One such study attempted to use VR to examine the effect of visual field motion on standing postural control within 3 days of SRC and reported that this VR perturbation caused motion sickness, dizziness, and disorientation acutely post SRC [17]. Therefore, subsequent studies focused on sub-acute recovery from SRC rather than acute diagnosis. These studies were able to identify differences in postural control and neuropsychological performance when other clinical assessments suggested complete recovery.

**Table 1:** Studies using VR in sport-related concussion management.

Study	Concussion cohort	Timepoints	VR assessment	Findings
Slobounov* et al.,* 2006 [17]	10 collegiate athletes	Baseline, days 3, 10, 30 following concussion	“Moving room” postural control assessment with AP translation and lateral roll	Subjects were unable to maintain postural control while viewing the “moving room” on day 3 post-injury; coupling between visual motion and postural control was diminished on day 10 post-injury
Slobounov* et al.,* 2006 [24]	8 collegiate athletes	Baseline, days 3, 10, 30 following concussion	“Moving room” postural control assessment with AP translation and lateral roll	Postural control deficits induced by visual field motion were present up to 30 days post-injury
Slobounov* et al.,* 2007 [25]	38 collegiate athletes, 9 experienced a second concussion within 1 year	Baseline, days 10, 17, 30 following concussion	“Moving room” postural control assessment with AP translation and lateral roll	Rate of recovery of sensorimotor integration was slower after the second concussion
Nolin* et al.,* 2012 [34]	25 adolescent athletes with concussion, 25 adolescent athletes with no concussion	Within 2 years following concussion	ClinicaVR: Classroom-CPT	The concussion group performed worse on the virtual version of the CPT but not on the computerized version
DeMatteo* et al.,* 2014 [33]	24 youth (ages 9-18 years)	Within 1 year following concussion	Nintendo Wii Ultimate Party Challenge and Wii Fit	Wii Gameplay was not a valid measure of postural control following concussion
Murray* et al.,* 2014 [31]	9 athletes with concussion, 9 athletes with no concussion	Within 48-72 h following concussion	Nintendo Wii Fit Soccer Heading Game	The concussion group had a greater number of Gaze Deviations from center than control group. There were no group differences for Percentage Time on Center or Soccer Game Score
Teel and Slobounov, 2015 [26]	28 collegiate athletes with concussion, 94 college students with no concussion	Within 7-10 days following concussion	HeadRehab postural control testing	The concussed group performed worse on all measures
Teel* et al.,* 2016 [27]	27 collegiate athletes with concussion, 94 collegiate athletes with no concussion	Within 7-10 days following concussion	HeadRehab postural control testing	The postural control module has high sensitivity (85.7%) and specificity (87.8%)
Teel* et al.,* 2016 [35]	24 collegiate athletes with concussion, 128 collegiate athletes with no concussion	Within 7-10 days following concussion	HeadRehab neuropsych. testing	Spatial navigation (sensitivity 95.8%/specificity 91.4%), whole body reaction time (sensitivity 95.2%/specificity 89.1%) and combined VR modules (sensitivity 95.8%/specificity 96.1%) had high sensitivity/specificity
Murray* et al.,* 2017 [32]	56 collegiate athletes with concussion, 79 physically active college students with no concussion	Within 24-48 h following concussion	Nintendo Wii Fit Basic Balance Test	The total number of levels completed (sensitivity 39.2/specificity 82.1%), time to complete level 1 (sensitivity 87.5%/specificity 25.3%), and level 5 completion (sensitivity 80.4%/specificity 39.2%) had weak predicative capability (AUC<0.7)
Wright* et al.,* 2017 [30]	11 collegiate athletes with concussion, 56 physically active college students with no concussion	Within 3 months following concussion	VETS	The concussion group performed worse on the VETS. The VETS had 91.0% accuracy and ROC AUC of 0.865
Wright* et al.,* 2017 [29]	14 college students with concussion, 58 college students with no concussion	Within 6 months following concussion	VETS	The dynamic scene conditions, i.e., DYN-Foam and DYN-Firm, were the most discriminating conditions with and 85.9% and 87.3% accuracy, respectively

VR: Virtual reality, SRC: Sport-related concussion, ER: Extended reality, AR: Augmented reality, MR: Mixed reality, ROC: Receiver operating characteristic, AUC: Area under the curve

### 2.1. Physical assessments

The “moving room” paradigm is a classical experimental system for the study of perceptual-motion integration by observing postural sway induced by optic flow [18-23]. In the “moving room” paradigm, the participant stands in a fixed inertial frame (i.e., on the ground), and the visual environment moves relative to this inertial frame (e.g., actually moving the walls of a mock room or providing a visual display that simulates such movement). The results of such experiments suggest that visual motion can induce postural sway in the direction of the visual motion, particularly when the visual motion is observed at low frequencies (e.g., 0.2 Hz) [18-23]. Using this “moving room” paradigm in VR, Slobounov *et al*. demonstrated the destabilizing effect of visual field motion following SRC [17,24]. Specifically, participants were unable to maintain postural control while viewing the “moving room” on day 3 following SRC and experienced motion sickness, dizziness, and disorientation [17]. Furthermore, there was an increase in the center of pressure (COP) area and a decrease in COP coherence up 30 days following SRC [17,24]. COP coherence is a measure of the relationship between scene movement and body sway. Higher COP coherence suggests that subjects more closely match their postural responses to scene movement. Thus, the observed decrease in COP coherence suggests perceptual-motion disintegration induced by visual field motion. All participants were asymptomatic by day 10 and were cleared for return to play, and COP area without visual motion was unchanged relative to baseline. The rate of recovery of sensorimotor integration was even slower after a second SRC [25].

Teel *et al*. used a clinical version of this VR-based “moving room” paradigm (i.e., HeadRehab) with postural control scores on a scale from 0 to 10 (10 is the best possible performance) [26]. A cutoff score of 8.25 had 85.7% sensitivity and 87.8% specificity (area under the curve [AUC]=0.862) in differentiating SRC from control within 7-10 days following SRC [27], whereas current clinical assessments have worse psychometric properties. For example, a balance error scoring system cutoff score of 21 had 60% sensitivity and 82% specificity (AUC=0.740) in differentiating SRC from control within 8 days following SRC [28].

Wright *et al*. also reported that visual field motion has a destabilizing effect following SRC using the virtual environment TBI screen (VETS) [29,30]. The VETS is a portable VR postural control device with testing done on both a firm and a foam surface, including static, no vision, and visual motion conditions. Within 3-6 months of SRC, participants had higher COP area and velocity than controls, particularly during the dynamic visual conditions (dynamic-foam had 85.9% accuracy and dynamic-firm had 87.3% accuracy in discriminating the SRC from the control group) [29,30].

Finally, three studies used out-of-the-box Nintendo Wii games, including Dinosaur, Arrows, Hamburger, Running, and Basketball as part of the Wii Ultimate Party Challenge, and Soccer Heading, Basic Run, and Basic Balance Test as part of the Wii Fit games [31-33]. DeMatteo *et al*. assessed postural control in 24 adolescents (mean age=14.9 years) within 1 year following SRC [33]. Wii postural control was not associated with community balance and mobility Scale or Bruininks-Oseretsky Test of Motor Proficiency – 2^nd^ Edition (BOT-2) scores [33]. Furthermore, most participants did not lose balance (62.5%) [33]. Although participants preferred Nintendo Wii games to traditional postural control measures, the Wii games were not a good measure for postural control in youth within 1 year following SRC [33]. It is important to note that the Nintendo Wii is a non-immersive VR system, which may have affected findings [4].

Murray *et al*. assessed postural control and oculomotor function within 72 h of SRC using both the Basic Balance Test and Soccer Heading [31,32]. The Wii Basic Balance Test provides real-time biofeedback about a participant’s COP. Participants are tasked to adjust their COP to a target area and maintain that position for 3 s. The Wii Basic Balance Test has five levels of difficulty that participants must complete within the 30 s allotted for the entire test. Outcomes include a total number of levels completed and time to complete each level. The concussion group completed fewer levels when compared to the control group and took longer to complete level 1, although no group differences were observed for time to complete levels 2-5 or total time to complete the test [32]. Furthermore, these outcomes had weak predictive capability in differentiating the concussion group from the control group (receiver operating characteristic [ROC] AUC=0.608 to 0.694) [32].

In addition to assessing postural control using the Wii Basic Balance Test, Murray *et al*. assessed the vestibulo-ocular reflex (VOR) using the Wii Fit Soccer Heading. The Wii Fit Soccer Heading tasks participants with heading a soccer ball while avoiding distractor objects. Eye movements were recording during testing with an eye-tracking system [31]. The concussion group had a greater number of Gaze Deviations from the center than the control group, but there were no group differences for Percentage Time on Center or Soccer Game Score [31]. These findings suggest a possible disruption in the VOR response during this activity due to either central sensory integration or peripheral vestibular deficits [31]. Together, these studies suggest that there is residual sensory integration dysfunction up to 30 days following SRC, but out-of-the-box and off-label uses of VR-based tools may not target outcomes of interest. Furthermore, non-immersive systems may be less sensitive in identifying SRC impairments.

### 2.2. Neuropsychological testing

Two studies have used VR-based tools for neuropsychological testing [34,35]. The ClinicaVR: Classroom-continuous performance test (CPT) is like the traditional VIGIL-CPT for sustained attention, vigilance, impulsivity, and reaction time except for the environment in which it is administered [34]. Specifically, in the VIGIL-CPT, single, and randomized letters are presented sequentially on a computer screen in white, on a black background; whereas, in the Classroom-CPT letters are presented on a blackboard in a virtual classroom in an immersive head-mounted display that features objects and people commonly found in real classrooms such as desks, a teacher, and students. Throughout the duration of the Classroom-CPT, the participant experiences auditory and visual distractions typical of a real classroom, such as a knock at the door, a bell announcing the end of class, children laughing outside, and a visit from the principal. Within 2 years following SRC, there were no differences between the SRC and control group in the traditional VIGIL-CPT, but the SRC group had more commission errors (i.e., they responded incorrectly) and more left-right head movement than the control group in the VR-based Classroom-CPT [34]. These findings suggest that participants had persistent deficits of attention and inhibition (i.e., they were unable to suppress their responses even if they were incorrect) and that the VR-based assessment approach was more sensitive to the effects of SRC than the traditional assessment [34]. One possible explanation is that the Classroom-CPT is more ecologically valid, thus placing greater demands on the participant’s capacities of attention and inhibition.

In addition to the postural control assessment, HeadRehab also includes neuropsychological assessments. Assessments can be administered on an immersive 3DTV or in a head-mounted display. Spatial memory is assessed with a navigational challenge through a virtual hallway. Whole-body reaction time is measured as the time in response to change in the direction of a “moving room.” Attention is examined by asking participants to count floors as they move up/down in a virtual elevator. The spatial navigation (sensitivity 95.8%/specificity 91.4%) and whole-body reaction time (sensitivity 95.2%/specificity 89.1%) tasks were able to discriminate the SRC from the control group (combined sensitivity 95.8%/specificity 96.1%) within 7-10 days following concussion [35]. The combined positive predictive value was 79.3% and the negative predictive value was 99.2% (AUC=0.989)[35]. These findings suggest that VR-based assessments may be more sensitive to SRC outcomes than traditional measures. The Classroom-CPT and HeadRehab neuropsychological assessments are immersive and closely represents real-life experiences, which may be more ecological and/or may place a greater demand on the participant’s cognitive function than pencil-and-paper and computerized neuropsychological assessments. Nonetheless, these studies are relatively small and findings must be replicated in larger, more diverse cohorts.

## 3. Discussion

As an emerging technology, VR is a promising assessment and rehabilitation tool for both researchers and health-care providers. This review aimed to determine the effectiveness of VR as an assessment tool for SRC. Despite small sample size and limited scope, these studies suggest that VR may be a more sensitive tool for SRC assessment [17,24-27,29,30,34,35]. VR-based postural control assessments suggest that visual motion is destabilizing following SRC [17,24-27,29,30]. VR can be used to create a “moving room” environment, which may induce a participant’s self-motion (i.e., egomotion). Egomotion has been attributed to the conflict between the moving visual stimulus and the vestibular and proprioceptive feedback [20]. When the visual stimulus does not match the other sensory feedback (i.e., somatosensory and vestibular), healthy young adults can reweight sensory feedback and ignore the destabilizing visual stimulus [36-38]. However, older adults and those with neurological impairments may be unable to adaptively switch between sensory modalities resulting in greater dependence on vision and higher sway when exposed to a visual stimulus [39,40]. Thus, this “moving room” paradigm can identify persistent perceptual-motion disintegration when clinical postural control tests suggest complete recovery [17,24]. Clinicians should be cautious; however, when using visual motion in the acute stages of SRC because VR may cause motion sickness, dizziness, and disorientation [17]. Persistent perceptual-motion disintegration may lead to subsequent musculoskeletal injury in the year following SRC [41]. Erroneous perceptions of environmental situations could result in unintentional musculoskeletal injuries [41]. Future VR-based rehabilitation interventions should further target sensorimotor integration.

Only two studies examined neuropsychological testing in VR following concussion [34,35]. However, both studies suggested that VR-based neuropsychological testing for SRC management is promising [34,35]. In comparison to traditional neuropsychological test batteries taken in a computer lab or clinic, VR can provide functional assessments using real-life scenarios or virtual environments such as the Classroom-CPT [34]. VR can range from low-tech video games like Nintendo Wii to high tech screen set-ups, which cover the entire visual field. Although the high tech screen set-ups may be too expensive or require too much space or technical expertise, VR-based neuropsychological testing using a head-mounted display is affordable and capable of testing the athlete on the sideline, in the locker room, in the clinic, or in the athletic training room. We discuss additional advantages and challenges briefly below. Several previous literature reviews further detail these topics albeit not specific to SRC [42-47].

### 3.1. Advantages

There are several advantages to VR-based SRC assessments and rehabilitation interventions [42-47]. For example, compared to the real world, a VR setting is highly controlled, customizable, and safe. However, compared to a traditional laboratory setting, a VR setting may be more ecologically valid; it more closely represents real-life experiences and provides multimodal and multisensory external stimuli. In addition, VR environments are expansive affording for complex sets of physical, cognitive, and behavioral functions rather than isolated components assessed by traditional measures. For both assessment and rehabilitation, VR can be remotely accessible, so the patient does not need to be in the same location as the health care provider to benefit from this type of technology. Gamification can motivate patients and biofeedback can bring attention to certain patient goals and provide feedback regarding their performance. VR also offers a form of escapism by immersing patients in another environment where they are free to explore safely their own limits. Finally, VR is becoming increasingly more accessible, less expensive, and more user friendly (e.g., head-mounted displays), which is ultimately needed to translate research findings into clinical practice.

### 3.2. Challenges

Despite the advantages presented above, there are also challenges that limit clinical practice from adopting VR [42-47]. For example, technical expertise is often required to set up and use VR-based assessment and rehabilitation tools. In addition, high fidelity systems can be prohibitively expensive, which can prevent clinics from being able to afford such systems. High fidelity systems can also be physically cumbersome, so space requirements may limit the types of technologies that clinics can adopt. These are challenges from the provider standpoint. There are also challenges from the patient standpoint. Specifically, non-veridical inputs can lead to misperceptions and sensorimotor distortions; individual differences in patient perceptions can cause adverse side effects (e.g., cybersickness); the long-term effects of VR on patient performance are often unknown. Finally, one of the biggest challenges with adopting VR technologies is that more high-quality research establishing validity, sensitivity, and specificity is required to inform evidence-based practice.

### 3.3. Emerging VR concussion management tools

Several companies make VR-based tools for SRC management. The goal of this review was not to provide the evidence for or against any of these devices, so health-care providers interested in incorporating VR in their clinical practice should ensure that these tools have sound psychometric properties and proven validity before adopting. Eye-Sync^®^ (SyncThink^®^, Palo Alto, CA) is a high-performance device that uses VR to assess abnormal eye movement. The device is portable and can be used in a clinical setting or outdoors. A person wears the VR head-mounted display and tracks a point of light to assess smooth pursuits, saccades, VOR, and VOR cancellation. Eye-Sync^®^ is the Food and Drug Administration approved to record and to analyze eye-tracking impairment. HeadRehab VR software (HeadRehab Inc., Chicago, IL) can be run on a 3DTV system or in a head-mounted display. HeadRehab VR software assesses spatial memory, postural control, reaction time, attention, and recall/recognition memory. Computerized dynamic posturography (CDP; Bertec, Columbus, OH) combines VR with force plates for assessment and rehabilitation of dizziness and postural control problems following SRC. Computer-assisted Rehabilitation Environment (CAREN; Motek, Amsterdam, The Netherlands) is a multisensory system for assessment and rehabilitation of sensorimotor impairments following SRC. These VR-based tools may be useful for health-care providers interested in incorporating VR in their clinical practice, but to be clinically useful, they must have sound psychometric properties and proven validity, and the tool must meet the desired outcome response. Therefore, clinicians should be cautious using out-of-the-box and off-label VR-based tools.

## 4. Conclusions

In the context of SRC assessment and rehabilitation, few studies have implemented VR solutions. Nonetheless, VR postural control and neuropsychological testing represent a promising next step in SRC management. VR allows for personalized interventions and may be more sensitive than traditional clinical assessments. However, VR must be implemented with caution because solutions must target specific domains. Each VR environment and application should be developed with a specific end user in mind; out-of-the-box or off-label VR-based tools may not target outcomes of interest. Clinicians should consider VR as another tool available to help in their assessment and rehabilitation protocols. Most VR programs are digitalization of existing assessments, but patient immersion in VR may result in improved ecological validity and controlled design and quantitative outcome measures may facilitate standardization. Although clinicians may be familiar with the standard version of these protocols, it is important to obtain training in the VR-based assessments. As with any new technology, it is important to identify the advantages and challenges for implementation. Ultimately, health-care providers trying to incorporate VR into clinical practice should consider applications best suited for their patients based on their symptoms to offer the best care possible.

### Conflicts of Interest

Fernando V. Santos, as an employee of Bertec Corporation, is developing products and has a financial interest related to the research described in this paper. No other authors have any conflicts of interest to disclose.
